# Corrigendum

**DOI:** 10.57264/cer-2024-0241

**Published:** 2025-01-07

The Research Article by John Sampalis *et al.*, ‘Real-world direct healthcare costs of treating recurrent high-grade serous ovarian cancer with cytotoxic chemotherapy’, which appeared in the June 2020 issue of the *Journal of Comparative Effectiveness Research* 9(8), 537–551 (2020), was published with an error in the affiliations.

The affiliations for Marwa Abo Alsoud appeared as:

^1^McGill University Health Center, Gynecologic Cancer Services, Cancer Care Mission, 1001 Decarie Boulevard, Montreal, QC, H4A 3J1, Canada

^2^McGill University, Department of Obstetrics & Gynecology, 1001 Decarie Boulevard, Montreal, QC, H4A 3 J1, Canada

The correct affiliations are:

^1^McGill University Health Center, Gynecologic Cancer Services, Cancer Care Mission, 1001 Decarie Boulevard, Montreal, QC, H4A 3J1, Canada

^2^McGill University, Department of Obstetrics & Gynecology, 1001 Decarie Boulevard, Montreal, QC, H4A 3 J1, Canada

^3^Clinical Oncology Department, Tanta University, Egypt

This has now been corrected.

The authors and editors of the *Journal of Comparative Effectiveness Research* would like to sincerely apologize for any inconvenience or confusion this may have caused our readers.

